# Compressive Mechanical Behavior of Partially Oxidized Polyvinyl Alcohol Hydrogels for Cartilage Tissue Repair

**DOI:** 10.3390/bioengineering9120789

**Published:** 2022-12-10

**Authors:** Silvia Todros, Silvia Spadoni, Silvia Barbon, Elena Stocco, Marta Confalonieri, Andrea Porzionato, Piero Giovanni Pavan

**Affiliations:** 1 Department of Industrial Engineering, University of Padova, via Venezia 1, 35131 Padova, Italy; 2 Department of Neurosciences, Section of Human Anatomy, University of Padova, via A. Gabelli 65, 35121 Padova, Italy; 3 L.i.f.e.L.a.b. Program, Consorzio per la Ricerca Sanitaria (CORIS), Regione Veneto, via N. Giustiniani 2, 35128 Padova, Italy; 4 Fondazione Istituto di Ricerca Pediatrica Città della Speranza, Corso Stati Uniti 4, 35127 Padova, Italy

**Keywords:** polyvinyl alcohol, hydrogel, chemical oxidation, indentation, poroviscoelasticity, constitutive modeling

## Abstract

Polyvinyl alcohol (PVA) hydrogels are extensively used as scaffolds for tissue engineering, although their biodegradation properties have not been optimized yet. To overcome this limitation, partially oxidized PVA has been developed by means of different oxidizing agents, obtaining scaffolds with improved biodegradability. The oxidation reaction also allows tuning the mechanical properties, which are essential for effective use in vivo. In this work, the compressive mechanical behavior of native and partially oxidized PVA hydrogels is investigated, to evaluate the effect of different oxidizing agents, i.e., potassium permanganate, bromine, and iodine. For this purpose, PVA hydrogels are tested by means of indentation tests, also considering the time-dependent mechanical response. Indentation results show that the oxidation reduces the compressive stiffness from about 2.3 N/mm for native PVA to 1.1 ÷ 1.4 N/mm for oxidized PVA. During the consolidation, PVA hydrogels exhibit a force reduction of about 40% and this behavior is unaffected by the oxidizing treatment. A poroviscoelastic constitutive model is developed to describe the time-dependent mechanical response, accounting for the viscoelastic polymer matrix properties and the flow of water molecules within the matrix during long-term compression. This model allows to estimate the long-term Young’s modulus of PVA hydrogels in drained conditions (66 kPa for native PVA and 34–42 kPa for oxidized PVA) and can be exploited to evaluate their performances under compressive stress in vivo, as in the case of cartilage tissue engineering.

## 1. Introduction

Hydrogels are promising materials for tissue engineering (TE), able to mimic the mechanical behavior of biological tissues. Their structure is based on a continuous polymer network that can absorb and retain a large amount of water, higher than 90% [1,2]. The presence of absorbed water is one of the key factors providing excellent biocompatibility and mechanical behavior similar to one of the soft tissues [3].

Among synthetic hydrogels, polyvinyl alcohol (PVA) has been proposed as a scaffold for cartilage, skin, cornea, vascular, and cardiac TE [4,5]. In particular, PVA hydrogels have been widely investigated for the replacement of damaged cartilage due to their bi-phasic structure, similar to that of healthy cartilage, as well as their elastic, viscoelastic and compressive mechanical properties, associated also with a low friction coefficient [6,7]. As far as the application for articular cartilage repair is concerned, the analysis of PVA mechanical behavior under compressive load is of crucial importance.

Several studies investigated the compressive mechanical properties of hydrogels via unconfined [8,9,10] and confined compression tests [11], often associated with 3D digital image correlation technique to measure the strain distribution in situ [12,13,14,15]. Experimental data allowed to develop poroviscoelastic models able to describe materials composed of a porous viscoelastic polymer matrix and an aqueous phase that can flow out due to loading and flow in over time [16,17]. The presence of a fluid phase can affect to a great extent the mechanical behavior of the hydrogel in compressive mode [18] and the typical time-dependent mechanical response shown in this condition [8,19]. Experimental studies in the literature adopted different drained and undrained experimental conditions, and therefore the estimated values of Young’s modulus were found to differ by more than 50% depending on the adopted experimental method [8,9,20]. Moreover, the mechanical properties of PVA hydrogels are known to be extremely sensitive to the preparation conditions, including PVA molecular weight, percentage weight of PVA in water, and cross-linking method. In particular, in the case of the freeze-thawing (FT) method, the mechanical behavior strongly depends on the time and temperature of freezing and thawing and on the number of FT cycles [21]. This is due to the fact that type (ionic/covalent), number, and length of cross-linking in hydrogels can dramatically affect their elastic modulus, with the stronger cross-linking generally associated to hydrogel stiffening and embrittlement [22].

Currently, in the literature, there is a wide range of approaches aimed at developing hydrogels with tunable stiffness and strength for TE applications, even if there is still no consensus on the best way to improve their mechanical properties. Tuning the stiffness from low to high values, hydrogels have been proposed for viscosupplementation [23,24], as scaffolds for cartilage TE [25] and repair of osteochondral defects [26]. Ideally, hydrogel composition and structure should mimic the native cartilage tissue, being able to withstand mechanical stress, especially in load-bearing joints. Among several approaches adopted to tune the mechanical properties, partial oxidation of PVA has been proposed through a chemical reaction with different oxidizing agents, namely potassium permanganate (KMnO_4_), bromine (Br_2_), and iodine (I_2_) [27,28,29]. Partially oxidized PVA hydrogels demonstrated high biocompatibility, good protein delivery capacity, and improved biodegradability in both in vitro and in vivo studies testing the polymer material not only for cartilage substitution [27], but also for peripheral nerve [30] and gut [31] TE. Regarding the mechanical behavior, uniaxial tensile tests showed that the oxidation treatment reduces PVA stiffness to a different extent depending on the oxidizing agent. This suggests the possibility to tune the mechanical properties according to different applications in vivo [30]. Moreover, the time-dependent response of oxidized PVA, investigated through stress relaxation tests, was found to be comparable to that of native PVA and was modeled as a quasi-linear viscoelastic behavior [32].

In cartilage TE, the mechanical performance of the substitute biomaterial under compression forces appears to be fundamental to withstand the complex loading environment of the joint in the perspective of in vivo implant [7,33]. Indentation tests have been widely used for measuring the compressive mechanical properties of polymers [14,15,34], hydrogels [35,36,37], and articular cartilage [38] at different length scales, by changing the size of the indenter. Since the compressive mechanical behavior of partially oxidized PVA hydrogels has not been studied yet, the aim of this work is characterizing native and oxidized PVA by means of indentation and consolidation tests, considering both almost-instantaneous and time-dependent mechanical response. The mechanical behaviors were described using a poroviscoelastic model and the associated material constants were obtained by fitting the FE results to the experimental data using an optimization method.

## 2. Materials and Methods

### 2.1. Preparation of PVA and Oxidized PVA Hydrogels

An aqueous solution of 16% wt PVA (Molecular Weight (MW) 124,000–184,000 Da) was prepared as already described by [28]. Partially oxidized PVA was obtained using different oxidizing agents, i.e., KMnO_4_ in dilute HClO_4_, Br_2_ in NaHCO_3_ buffer solution, and I_2_ in NaHCO_3_ buffer solution, according to well-consolidated protocols [28,30]. Based on the oxidizing agent used, partially oxidized PVA is referred to as OxPVA_KMnO_4_, OxPVA_Br_2_, and OxPVA_I_2_ from here on.

Hydrogels were prepared by pouring the solution of native or oxidized PVA into cylindrical molds (internal diameter 22 mm, height 7 mm). Physical cross-linking was achieved through the FT method [21], with three cycles including freezing at −20 °C for 24 h and thawing at −2.5 °C for 24 h. The hydrogels were then stored at −20 °C until use. A detailed description of the preparation and characterization of oxidized PVA hydrogels was reported in previous works [28,30].

### 2.2. Mechanical Tests

Mechanical tests were run on a Bose ElectroForce^®^ Planar Biaxial Test Bench instrument (TA Instruments, New Castle, DE, USA).

**Figure 1:**
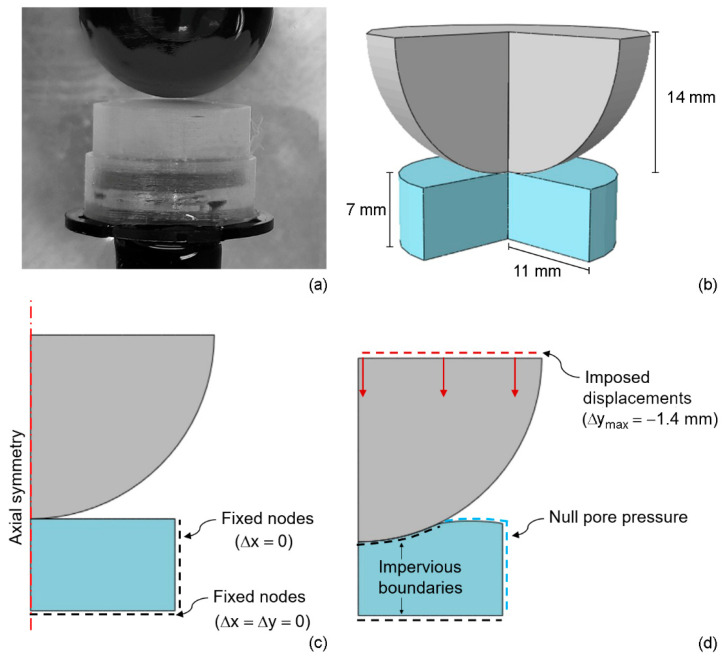
(**a**) Experimental setup for indentation and consolidation tests: detail of sample surface and indenter before testing. (**b**) Numerical model: cut-out view of the 3D geometry with size of sample and indenter; (**c**) axisymmetric FE model adopted for the analyses with indication of fixed displacement boundary conditions; (**d**) imposed displacements and fluid phase boundary conditions.

The samples were unfrozen about 1 h before any experiment by immersing them in physiologic solution at room temperature and stayed hydrated in submerged condition for the overall duration of the tests. Samples were kept in cylindrical molds, having also the function of sample holders. Indentation tests were performed by using a rigid spherical indenter with a diameter of 28 mm (Figure 1a).

The indenter was slowly lowered onto the hydrogel surface up to contact, which is assumed when measuring a compressive force equal to −0.005 N. Tests were then performed at an indentation depth rate of 10 mm s^−1^, up to −1.4 mm. This indentation depth corresponds to 20% of the overall sample height and was selected according to the physiological range of maximum compressive strain for human cartilage [39,40] and to the strain range previously adopted in tensile mode [32]. Consolidation tests were carried out by keeping constant compressive strain for 5400 s. Each sample was used for a single test and five samples were tested for each material.

### 2.3. Swelling Tests

The analysis of the swelling behavior of native and oxidized PVA hydrogels allows to measure the amount of water that flows in the hydrogel pores in an aqueous environment without dissolution, and to determine the void ratio in the polymer matrix. For this purpose, 3 samples of each PVA hydrogel (5 × 2 × 30 mm) were separately immersed in 5 mL of phosphate-buffered saline (PBS) 1× solution, at room temperature and 95% relative humidity for a total time of 600 h. Every 24 h, PBS excess was removed from the hydrogel surface by wiping it with filter paper, then each sample was weighed. The swelling percentage was calculated using the following equation:

(1) $$swelling\;\left(\%\right)=\frac{W_{s}-W_{d}}{W_{d}}\cdot 100$$

where $W_{s}$ is the weight of the swollen hydrogel and $W_{d}$ is the weight of the dry hydrogel.

The void ratio is defined as:

(2) $$e_{0}=\frac{V_{w}}{V_{m}}$$

where $V_{w}\;$ is the volume of water that can flow in the pores and $V_{p}\;$ is the volume of the solid polymer matrix. In case of a swollen hydrogen, this expression can be written as:

(3) $$e_{0}=\frac{\left(W_{s}-W_{d}\right)\cdot \rho_{w}}{W_{d}\cdot \rho_{p}}$$

where $\rho_{w}$ is the water density and $\rho_{p}\;$ is the density of the polymer in the solid matrix. The density of PVA polymer matrix, obtained from aqueous solutions with PVA concentration up to 16 wt%, is reported to be in the range 1–1.03 g/cm^3^ [41,42]. Considering that this value is close to water density, the void ratio can be assumed equal to the swelling ratio (i.e., swelling percentage/100).

### 2.4. Constitutive Models

An isotropic poroviscoelastic model was used to account for both the solid and the liquid phases of native and oxidized PVA hydrogels. Even the water flow across the hydrogel matrix was considered, assuming a bi-phasic system under the hypothesis of a fully saturated medium. The permeability of the matrix must be provided, in particular ABAQUS CAE/ABAQUS Standard (SIMULIA™, Dassault Systems, Velizy-Villacoublay, France) requires the specific permeability, which is defined as $k_{s}=\gamma_{w}\cdot k$, where $\gamma_{w}$ is the specific weight of the wetting liquid, in this case water, which is equal to 9.81 × 10^−6^ N/mm^3^ and k is the absolute permeability. The values for the specific permeability were determined through an optimization procedure performed in MATLAB (MathWorks, Natick, MA, USA) and assumed to be constant with the void ratio. The values for the void ratio were estimated from swelling measurements as above mentioned.

The constitutive models for the solid phase of native and oxidized PVA hydrogels were developed under the hypothesis of linear viscoelastic behavior. This assumption was made considering that the stress-strain response in tensile behavior of native and partially oxidized PVA was found almost-linear up to a nominal strain of 20% in a previous work [33]. Therefore, this behavior was assumed also in compression.

The elastic properties, at drained conditions, are defined by the longitudinal elastic modulus E and the Poisson’s ratio ν. The viscoelastic response is due to the deviatoric part of the stress tensor $\tilde{\sigma }$, which is given by:

(4) $$\tilde{\sigma }=\tilde{\sigma_{0}}-\overset{m}{\underset{i=1}{\sum }}\frac{\gamma_{i}}{\tau_{i}}\int_{0}^{t}exp\left(-\frac{t}{\tau_{i}}\right)\;F^{-1}\left(t-s\right)\tilde{\sigma_{0}}F\left(t-s\right)ds$$

where m is the number of the visco-elastic branches, equal to four, and $\gamma_{i}$, $\tau_{i}$ are the material constants defined as relative stiffness and relaxation times, respectively and ***F*** the deformation gradient [43,44]. The material constants for the viscoelastic response of native and oxidized PVA were determined in a previous study on the same hydrogels [32] and assumed to be valid also in the compressive range of the solid phase (Table 1). The parameters responsible for the long-term elastic response of the PVA specimens, together with the permeability, were computed through an optimization procedure, as described below.

### 2.5. Numerical Analyses

Finite element (FE) axisymmetric numerical models of PVA samples and indenter were developed by means of ABAQUS CAE/ABAQUS Standard to replicate the experimental setup (Figure 1b).

In the real scenario, the PVA sample is fixed on the bottom of the sample holder. In the model, the nodes on the endplate were not allowed to move in any direction while the movement of the nodes on the lateral surface was prohibited in radial direction only (Figure 1c).

Numerical analyses were carried out applying the same loading condition of experimental tests (Figure 1d), thus by imposing a displacement of 1.4 mm perpendicular to the hydrogel disk surface. The friction coefficient between the spherical indenter and the PVA sample was chosen after preliminary analyses where different values were considered. Since the different factors did not influence the results, a friction coefficient of 0.1 was assumed in the tangential direction, while a frictional contact was placed in the normal direction to avoid interpenetration.

Null pore pressure was assumed in the lateral face, due to the conditions offered by the cylindrical molds, and in the portion of the upper face of the sample where the indenter did not shove the surface (Figure 1d). The spherical indenter was modeled as a rigid body.

To mesh the PVA sample, an average size of 0.5 mm was used. To evaluate the fairness of this choice, even a mesh size of 0.25 mm was considered and the difference between meaningful acquired quantities was assessed with preliminary analysis. Since no relevant difference was found in the measured data, a mesh size of 0.5 mm was chosen to reduce the computational cost.

### 2.6. Constitutive Parameters Optimization

To identify specific permeability *k_s_* and the long-term parameters responsible for the hydrogel elastic response, namely Young’s modulus *E* and Poisson’s ratio *ν*, an inverse iterative procedure was used, combining ABAQUS Standard and MATLAB.

The objective function to be minimized was the mean squared error (*MSE*) of the reaction force over time between simulated data and mean experimental data measured on the indenter during the indentation-consolidation process:

(5) $$MSE\;\left(E,\;\nu ,\;k_{s}\right)=\frac{1}{N}\;\overset{N}{\underset{i=1}{\sum }}{\left[RF_{num,t_{i}}\;\left(E,\;\nu ,\;k_{s}\right)-RF_{exp,t_{i}\;}\right]}^{2}$$

where $RF_{num,\;t_{i}}\;\left(E,\;\nu ,\;k_{s}\right)$ and $RF_{exp,\;t_{i}\;}$ are the predicted and the experimental reaction force measured on the indenter, respectively.

To minimize the MSE between numerical and experimental data, a built-in MATLAB function was used, which finds the minimum of constrained nonlinear multivariable functions. A two-step procedure was applied. Firstly, the optimization was focused only on the long-term Young’s modulus E for native and oxidized PVA hydrogels. Therefore, only the indentation phase was examined: the optimal Young’s modulus E was computed in order to obtain a maximum value of the reaction force, measured at the end of the indentation phase (t = 0.15 s), not higher than the experimental value.

After calculating the long-term Young’s modulus *E* for all four PVA hydrogels, the inverse iterative procedure was run to find the optimal parameter for specific permeability k_s_ and long-term Poisson’s ratio *ν*.

The interval of calculation for the Poisson’s ratio was 0.45–0.49, chosen in agreement with [45], where the same variation range was measured, and with [46], where a mean value of Poisson’s ratio for PVA hydrogel of 0.449 with standard deviation of 0.031 was experimentally computed through an optical flow analysis.

A performance analysis test was conducted preliminarily to assess the capability of the optimization process using reference artificial data instead of experimental data.

### 2.7. Statistical Analyses

Experimental data were analyzed through Kruskal–Wallis nonparametric one-way analysis of variance (ANOVA) and post hoc comparison, considering as significant a *p*-value lower than 0.05. All the analyses were developed with MATLAB.

## 3. Results and Discussion

### 3.1. Indentation Tests

**Figure 2:**
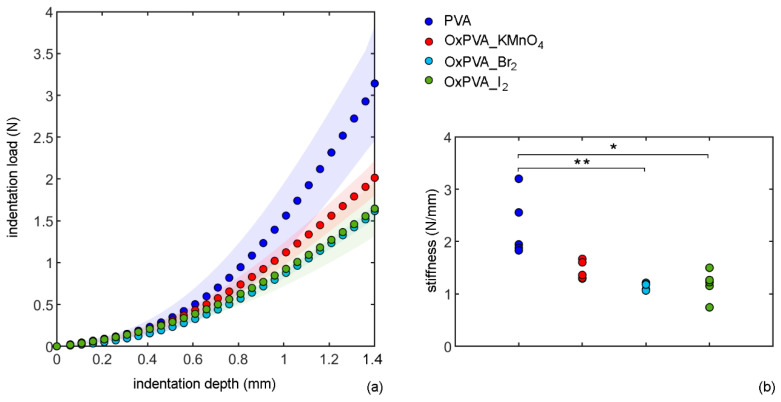
Results of indentation tests of native and oxidized PVA hydrogels: (**a**) indentation load (mean and confidence interval) vs. depth; (**b**) values of stiffness (N/mm) for each sample of native and oxidized PVA. Significant differences among hydrogels are indicated by one (*) or two (**) asterisks, respectively with *p*-value < 0.05 and *p*-value < 0.01.

The almost-instantaneous compressive responses of native and oxidized PVA can be compared in Figure 2, where the results of indentation tests are reported in terms of absolute values of both indentation load and depth. 

To compare the materials, the maximum absolute value of indentation load measured in each experimental test was divided by the maximum indentation depth (1.4 mm). This value represents a measure of stiffness. Examining the load-depth responses for the different samples, it may be noted that the maximum penetration depth was reached at a lower load for oxidized versus native PVA, suggesting a decrease in material stiffness after oxidation. However, a statistically significant difference was only calculated between PVA and OxPVA_Br_2_ (*p*-value = 0.002), and between PVA and OxPVA_I_2_, (*p*-value = 0.017). There is no statistically significant difference between the other materials.

Considering the physical properties of biomaterials, matrix stiffness is acknowledged as a fundamental cue to regulate cell behavior during cartilage regeneration [47]. Increasing the cross-linking degree and the mechanical stiffness of hyaluronic acid-based hydrogels was found to induce a shift in mesenchymal stem cells (MSCs) chondrogenesis toward the acquisition of a fibrous phenotype and the formation of fibrocartilage [48]. Additionally, modulating hydrogel composition and stiffness showed to influence chondrocyte proliferation and collagen/glycosaminoglycans production in vitro [49]. Based on these considerations, tunable mechanical stiffness of oxidized PVA under compression load could be functional to control cell–biomaterial interactions during chondrogenesis, modulating MSC/chondrocyte responses towards functional tissue regeneration.

### 3.2. Consolidation Tests

Consolidation is a phenomenon that occurs in highly hydrated biological tissues subjected to compressive loads, which cause fluid exudation and compaction of the solid phase [50]. The gradual loss of water from the tissue produces hydraulic lubricant and cushion effects which reduce friction and mitigate the impact of sudden loads in the tissue cavity or anatomical structures like the joint. Indeed, the consolidation process, applied to a one-dimensional confined compression loading scenario, has been proved to affect articular cartilage also [51]. Consolidation testing has recently been considered in biological contexts, for example, to assess the biomimetic features of fluid-saturated porous biomaterials like hydrogels in the perspective of TE application.

**Figure 3:**
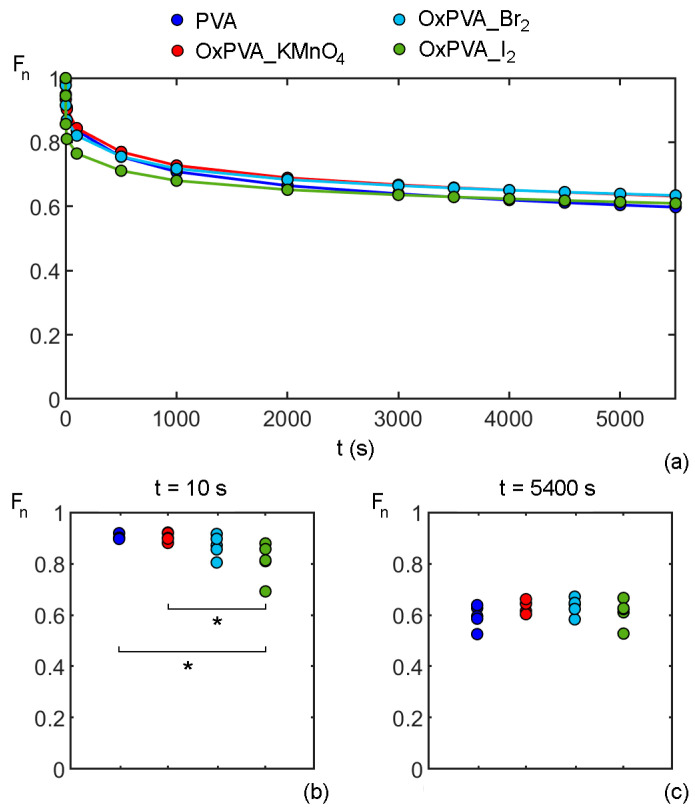
Results of consolidation tests of native and oxidized PVA hydrogels: (**a**) mean of normalized indentation force F_n_ vs. time; values of F_n_ for each sample of PVA hydrogels at different time instants, i.e., (**b**) 10 s and (**c**) 5400 s, during consolidation test. A significant difference among hydrogels is indicated by an asterisk (*) with *p*-value < 0.05.

Herein, the mechanical consolidation tests were performed on native and oxidized PVA to further characterize hydrogel response to compressive loads from the perspective of in vivo implant within the joint. For each consolidation test, normalized indentation force F_n_ was calculated as the ratio of force at the current time and the maximum force measured at the initial time of the consolidation process. The mean values of normalized force vs. time for each material, i.e., native and oxidized PVA hydrogels, are plotted in Figure 3a. Consolidation curves appear to be almost overlapped, except in the short-time range. In order to highlight possible differences in the consolidation process, normalized force values for the five experimental tests conducted for each material, were evaluated at two specific time instants to assess whether there is a statistically significant difference between the four tested materials through the indentation-consolidation process.

The two chosen times are 10 s (Figure 3b) and 5400 s (Figure 3c) to compare the four groups accounting for, respectively, the first relaxation phase and the asymptotic behavior during the consolidation process. At 10 s, a statistically significant difference was found between PVA and OxPVA_I_2_ (*p*-value = 0.038) and between OxPVA_I_2_ and OxPVA_KMnO_4_ (*p*-value = 0.028). At 5400 s, no differences were observed among the four materials: a similar asymptotic behavior was shown by all PVA hydrogels, exhibiting a force reduction of nearly 40% from the initial time instant of the consolidation process.

This time-dependent mechanical behavior is slightly different from the one of articular cartilage in compressive conditions, where a force reduction of about 60% [52,53] has been measured. However, the use of different experimental setup in the literature makes it difficult to compare results on PVA hydrogel and animal/human articular cartilage.

### 3.3. Swelling Behavior

Swelling is one of the key properties of hydrogel biomaterials. This is due to their capacity to retain a significant fraction of water within their polymeric structure when in contact with an aqueous solution or a biological fluid. Changes in the swelling behavior may reflect the hydrogel responsiveness to intrinsic or external modifications. From a biological point of view, the ability to swell is related to hydrogel capacity to load protein solutions and thus act as biomolecule/drug delivery systems [28].

**Figure 4:**
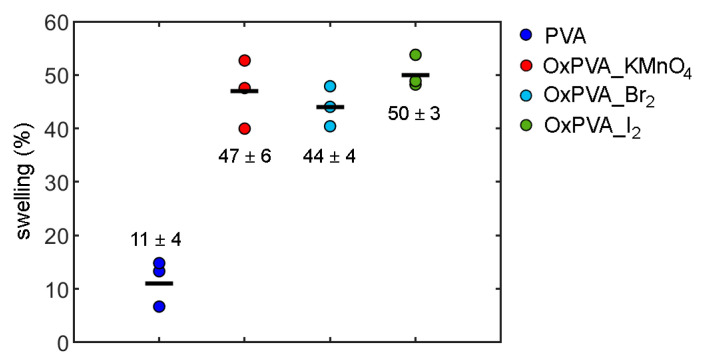
Swelling % for native and oxidized PVA hydrogels at equilibrium in PBS at 37 °C. For each material, the swelling ratio mean values (black dash) and the standard deviation are indicated.

Figure 4 describes the swelling of native and oxidized PVA hydrogels after 600 h of incubation in PBS, when equilibrium conditions are reached. Indeed, when a porous PVA polymer matrix is in contact with an aqueous solution, the network starts to swell due to water absorption.

The swelling force, due to the interaction between polymer chains and water, is balanced by a shrinking force induced by the crosslinks of the polymer matrix.

When these forces are equal, swelling equilibrium is reached. While native PVA shows an equilibrium swelling of about 11%, oxidized PVA hydrogels are able to retain a much higher amount of water and present similar swelling % values. These data are adopted in the constitutive model to quantify the different void ratios (equal to swelling percentage/100) in native and oxidized PVA hydrogels.

### 3.4. Constitutive Modeling

**Figure 5:**
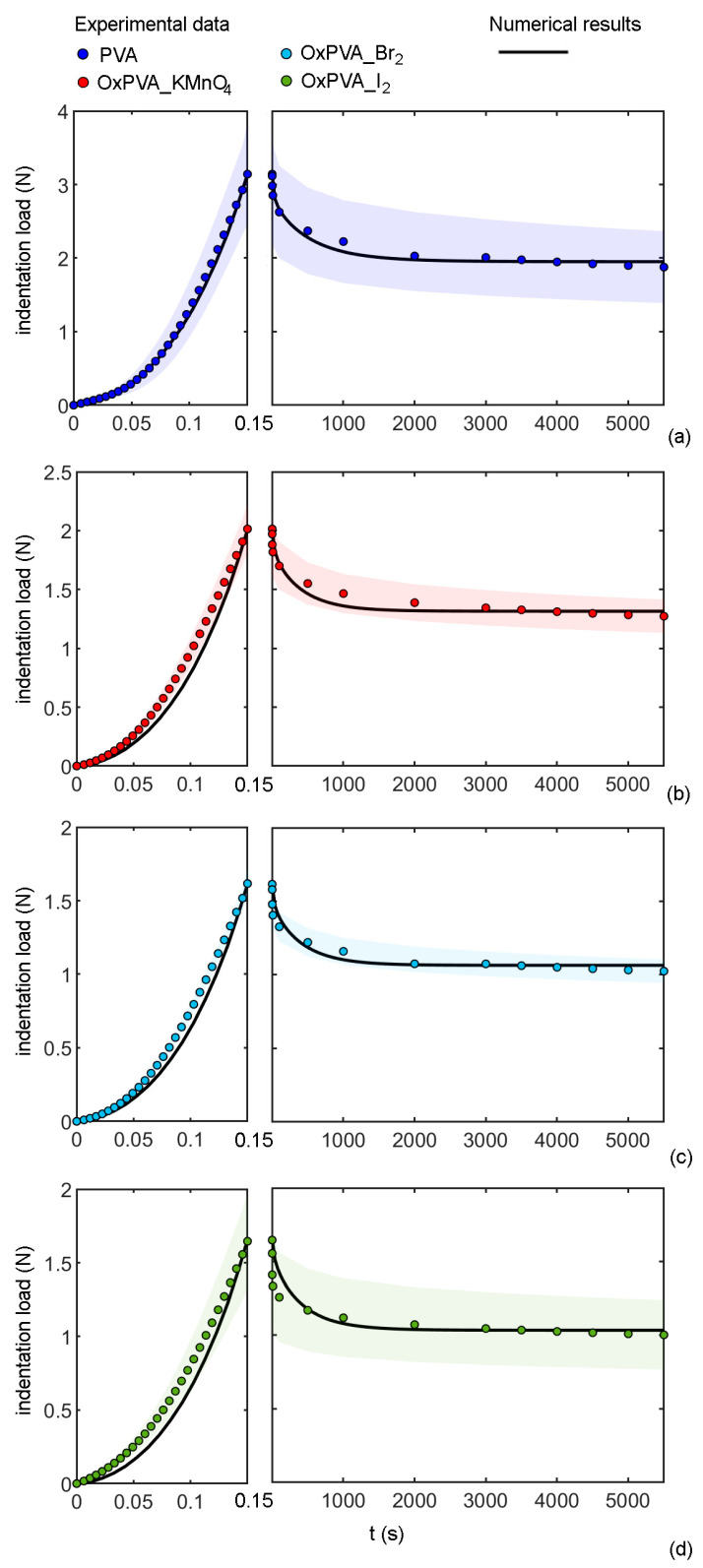
Comparison between experimental data and numerical results of indentation and consolidation tests for native and oxidized PVA hydrogels: indentation load (mean and confidence interval) vs. time for (**a**) PVA, (**b**) OxPVA_KMnO4, (**c**) OxPVA_Br_2_, and (**d**) OxPVA_I_2_.

The comparisons between experimental data and numerical fitting are shown in Figure 5 and the optimal model parameters are reported in Table 2.

As described above, the fitting was performed by minimizing the mean squared error (MSE) between the experimental data and the fitted numerical data, therefore this value can be considered an indicator of the accuracy of the combined fitting procedure. For the four tested materials, the obtained MSE were 3.2 × 10^−3^ for PVA, 2.8 × 10^−3^ for OxPVA_KMnO_4_, 6.0 × 10^−4^ for OxPVA_Br_2_, and 1.9 × 10^−3^ for OxPVA_I_2_. All the obtained MSE suggested a good correspondence between the numerical and the experimental results.

**Figure 6:**
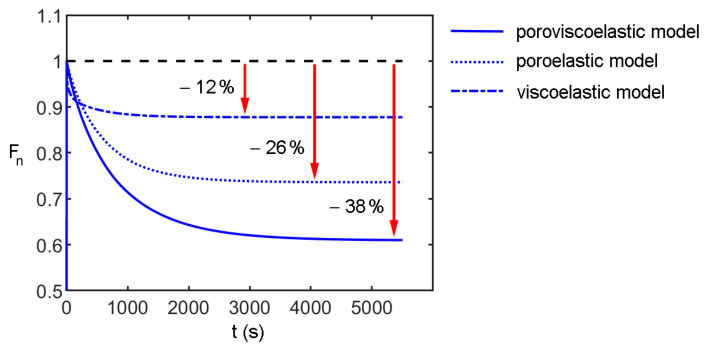
Comparison of the effects of different constitutive models on the consolidation behavior: the major reduction of normalized indentation force occurs at different characteristic times and with different variations marked by red arrows.

The time-dependent behavior of PVA hydrogels in compression is due to the contribution of the viscoelastic behavior of the polymer matrix and the water flow through the hydrogel pores. To point out the effect of these contributions, numerical simulations were carried out using the optimal constitutive parameters determined for native PVA and comparing the hypothetic behavior of a viscoelastic and a poroelastic model (therefore, excluding the migration of fluid phase and the viscous effects, respectively) with the adopted poroviscoelastic model. The results are summarized in Figure 6 in terms of normalized indentation force over time. It is evident that the viscoelasticity accounts only for 12% of the total drop of the indentation force, while the effects of the migration of the fluid phase account for 26%, for a total drop of about 38%. Moreover, the time-dependent effects associated with viscoelasticity are faster than the time-dependent behavior associated with the migration of fluid phases. In fact, based on the viscoelastic constants of Table 1, after the first 20 s of consolidation, the rate of indentation force drop is below 0.2% s^−1^. This explains the necessity to assume a poroviscoelastic model to properly describe the overall behavior of PVA in compression.

The values of Young’s modulus reported in Table 2 can be related to the compressive stiffness of the articular cartilage, reported in the range 0.1–2.0 MPa in different loading conditions (confined/unconfined compression or indentation) on animal models or humans in different joints [40]. It should be considered that the moduli obtained from numerical modeling represent the long-term stiffness of native and oxidized PVA hydrogels in drained conditions, while the results reported for articular cartilage are generally almost-instantaneous measurements of stiffness. In this sense, the obtained Young’s moduli of PVA hydrogels can be considered compatible with the lowest values of cartilage compressive stiffness in the literature and thus small PVA patches may be suitable for the use for the repair of cartilage defects, from a biomechanical point of view.

**Figure 7:**
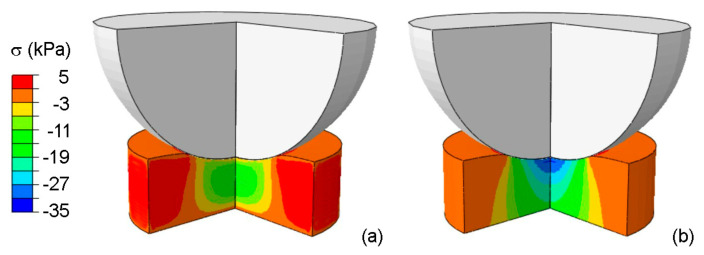
Contour of true stress σ (kPa) in the indentation direction at the end of (**a**) indentation and (**b**) consolidation of native PVA.

To highlight the effects of stress distribution over time during consolidation, the contour of true stress in the indentation direction is reported in Figure 7, both at the end of indentation and at the end of consolidation (corresponding respectively to a time of 0.15 s and 5400 s in Figure 5). As expected in a bi-phase material, in the instantaneous initial loading, the stress results are more homogenous with respect to the final consolidated state corresponding to the equilibrium in the fluid phase movement.

Despite the satisfactory results obtained in the simulation of indentation and consolidation tests, the constitutive model adopted could be further improved, and some limits of the proposed approach should be considered. Firstly, the drained behavior of both native and oxidized PVA hydrogels is considered linear (in terms of Cauchy stress vs. logarithmic strain). This assumption agrees with other works in the literature [8]; on the other hand, also, the tensile behavior found in previous work [32] shows an almost linear behavior up to the nominal strain of 20%. Compressive tests at drained conditions, i.e., characterized by a very low strain rate, should be developed to better investigate this aspect. The viscoelastic behavior of the polymeric matrix in compression was assumed to be equal to the one identified with the tensile tests. This assumption could be confirmed by including the set of the viscoelastic parameters (relaxation moduli and time constants) in the free parameters of the optimization process. However, this would complicate the procedure and the capability of convergence to an optimal solution.

Moreover, an additional characterization of the microstructure and porosity of PVA hydrogels could be carried out to determine the void ratio by means of different methods. For example, cryogenic Scanning Electron Microscopy (cryo-SEM) is often adopted to determine the pore size through image analysis [54,55]; however, cryo-SEM does not allow to accurately determine the total volume of porosity in a bulk sample. Also, the relaxation times measured via nuclear magnetic resonance (NMR) in hydrogels have been used to obtain the pore radius distribution profiles [56]. The analysis of the pore size in PVA hydrogels for TE is fundamental not only to evaluate the effect of water flow during compressive loading, but also possible modifications induced after cell seeding as because of cellular growth and proliferation within the matrix [57]. Indeed, pore size plays an important role in the signaling and microenvironmental stimuli provided to the cells.

## 4. Conclusions

Extensive research activities are currently focusing on the design of biomaterials for articular cartilage restoration. However, it is still difficult to develop implant substitute materials able to function synergistically with healthy cartilage. In this context, hydrogels have emerged as one of the most promising types of polymers to mimic cartilaginous tissue, with several studies investigating strategies to finely control and tune their biomechanical properties. Among hydrogels, PVA shows adjustable chemical and mechanical features, and its surface bioactivity can be increased through functionalization. Nonetheless, the characterization of PVA biomechanics has not been extensively focused on articular cartilage replacement yet.

In this work, the effect of different oxidizing agents on the compressive mechanical behavior of native and partially oxidized PVA hydrogels was investigated, through indentation and consolidation tests considering both almost-instantaneous and time-dependent mechanical responses. Experimental results showed that the oxidizing treatment reduces the compressive stiffness of PVA during indentation, while the consolidation curves were not significantly affected by oxidation, except in the short-time range. A different swelling behavior was observed after partial oxidation, since native PVA showed an equilibrium swelling of about 11%, while oxidized PVA hydrogels were found to be able to retain a much higher amount of water, with mean swelling values between 44% and 50%. This modification can be considered beneficial for cartilage repair, since the hydration conditions of partially oxidized PVA hydrogels are closer to those of healthy articular cartilage.

A poroviscoelastic constitutive model was developed to describe the time-dependent mechanical response of native and partially oxidized PVA hydrogel. The contribution of the viscoelastic polymer matrix and the flow of water molecules within the matrix during long-term compression were considered and a good correspondence between numerical and experimental results was reached. The long-term Young’s modulus of PVA hydrogels in drained conditions was estimated through constitutive modeling and the resulting values (66 kPa for native PVA and 34–42 kPa for oxidized PVA) can be considered close to cartilage elastic modulus. Indeed, other studies in the literature reported values of almost-instantaneous modulus of articular cartilage equal to about 100 kPa and a stiffness decrease up to 60% has been measured in persistent compressive conditions over time. A more in-depth comparison between the compressive behavior of PVA hydrogels and articular cartilage will be carried out by testing human knee articular cartilage with the same indentation and consolidation protocols adopted in this work.

At this stage, the obtained results indicate that native and partially oxidized PVA hydrogels have a mechanical behavior similar to the one of cartilage tissues, therefore they are good candidates for replacement materials. As a future perspective, understanding the effects of substrate stiffness on chondrocyte phenotype and functions, as well as on stem cell chondrogenesis, would improve biomaterial design for cartilage TE applications.

Despite some limitations of the study, this poroviscoelastic constitutive model, with optimized parameters depending on the type of PVA hydrogel, would allow the evaluation of the time-dependent compressive response of these biomaterials in different applications, especially in articular cartilage repair. This may be obtained by means of numerical analyses reproducing the loading condition in vivo and patient-specific geometry of cartilage defects to be repaired.

## Figures and Tables

**Table 1:** Relaxation moduli and time constants of native and oxidized PVA samples.

	γ_1_	τ_1_ (s)	γ_2_	τ_2_ (s)	γ_3_	τ_3_ (s)	γ_4_	τ_4_ (s)
PVA	0.020	0.523	0.039	5.00	0.044	50.0	0.057	500.0
OxPVA_KMnO_4_	0.016	0.538	0.025	5.00	0.043	50.0	0.082	500.0
OxPVA_Br_2_	0.009	0.439	0.029	4.93	0.042	50.0	0.079	499.8
OxPVA_I_2_	0.009	0.560	0.023	4.98	0.037	50.0	0.085	500.1

**Table 2:** Optimal parameters: Young’s modulus, Poisson’s ratio, and specific permeability.

	E (KPa)	ν	k_s_ (N/mm)
PVA	66	0.46	5.5 × 10^−7^
OxPVA_KmnO_4_	42	0.47	1.0 × 10^−6^
OxPVA_Br_2_	34	0.47	1.2 × 10^−6^
OxPVA_I_2_	35	0.46	1.4 × 10^−6^

## Data Availability

The data presented in this study are available on request from the corresponding author.

## Abbreviations

The following abbreviations are used in the manuscript:

TE	Tissue engineering
PVA	Polyvinyl alcohol
FT	Freeze-thawing
MW	Molecular weight
OxPVA_KMnO4	PVA partially oxidized with KMnO4
OxPVA_Br2	PVA partially oxidized with Br2
OxPVA_I2	PVA partially oxidized with I2
PBS	Phosphate-buffered saline
FE	Finite element
MSE	Mean squared error
ANOVA	Analysis of variance
MSCs	Mesenchymal stem cells
Cryo-SEM	Cryogenic Scanning Electron Microscopy
NMR	Nuclear magnetic resonance
